# Histomorphological Effects of Repeated Administration of the TRPA1 Antagonist HC-030031 on Intrafusal and Extrafusal Muscle Fibers in Healthy Young Rats

**DOI:** 10.3390/muscles5030051

**Published:** 2026-07-17

**Authors:** Takahiro Furumi, Ryoya Oga, Hiroyuki Tamaki

**Affiliations:** Department of Sports and Life Science, National Institute of Fitness and Sports in Kanoya, 1 Shiromizu, Kanoya 891-2393, Kagoshima, Japan; furumi0002@gmail.com (T.F.); m247001@sky.nifs-k.ac.jp (R.O.)

**Keywords:** TRPA1 antagonist, muscle spindle, skeletal muscle histomorphology

## Abstract

Transient receptor potential ankyrin 1 (TRPA1) is a non-selective cation channel expressed in primary sensory neurons and has been proposed as a therapeutic target for pain management. However, the effects of repeated administration of a TRPA1 antagonist on peripheral tissue morphology remain unclear. This study examined whether repeated HC-030031 administration affected the histomorphology of skeletal muscle fibers and muscle spindles in rats. Male Fischer 344 rats were assigned to a control group (Con, *n* = 10, 12.6 ± 3.3 weeks) or an HC-030031-treated group (HC, *n* = 10, 13.2 ± 3.7 weeks). HC-030031 was administered subcutaneously once weekly for four weeks. Tibialis anterior muscles were harvested and analyzed using histochemical and morphometric methods. Morphometric analyses included muscle mass, myofiber cross-sectional area (FCSA), roundness of extrafusal muscle fibers, NADH-TR staining intensity-based assessment, and structural parameters of muscle spindles and intrafusal fibers. No significant differences were observed between the groups in body weight, muscle mass, extrafusal FCSA, or fiber roundness. NADH-TR staining was used to distinguish fibers with darker or lighter staining intensity, and FCSA was compared between the Con and HC groups within each staining-based fiber subset. No group differences were detected in the FCSA of either subset. Similarly, no significant differences were observed in intrafusal FCSA, intrafusal fiber roundness, the number of intrafusal fibers per muscle spindle, or muscle spindle density. These findings indicate that repeated HC-030031 administration was not associated with detectable histomorphological alterations in skeletal muscle fibers or muscle spindles under resting conditions in young adult male rats.

## 1. Introduction

Transient receptor potential ankyrin 1 (TRPA1) is a non-selective cation channel predominantly expressed in small-diameter primary sensory neurons, including Aδ and C fibers [1]. TRPA1 functions as a polymodal sensor that responds to mechanical, chemical, and thermal noxious stimuli, and its activation contributes to nociceptive signaling and mechanical hyperalgesia under inflammatory or tissue-damaging conditions. In the context of skeletal muscle, sensory afferent signaling has been implicated in exercise-induced pain phenomena, including delayed-onset muscle soreness (DOMS) [2,3], which commonly occurs after unaccustomed or high-intensity exercise and can negatively affect training quality and athletic performance.

Pharmacological inhibition of TRPA1 has been shown to attenuate pain-related behaviors in various experimental models, including inflammatory, neuropathic, and chemically induced nociception [4]. TRPA1 antagonists reduce responses to cold stimulation and chemical irritants, such as allyl isothiocyanate [5], supporting the role of this channel in sensory hypersensitivity [6]. Accordingly, TRPA1 has been proposed as a promising therapeutic target for pain management. While previous investigations have provided valuable insights into behavioral outcomes and sensory signaling, the morphological consequences of repeated TRPA1 antagonist administration in target tissues are not well understood.

Despite increasing interest in sensory TRPA1 inhibition, little attention has been paid to its potential effects on peripheral tissues innervated by sensory neurons. Skeletal muscle is particularly relevant in this regard because it contains muscle spindles, specialized mechanosensory organs that contribute to proprioception and motor control [7].

Because TRP channels, including TRPA1, are involved not only in nociceptive signaling but also in the regulation of cellular and tissue homeostasis across multiple organ systems [8,9,10], it is important to examine whether repeated administration of a TRPA1 antagonist affects peripheral tissue morphology. Sustained modulation of sensory signaling could theoretically influence muscle trophism, fiber morphology, or spindle structure through changes in afferent activity [11]. This issue may be relevant when pain-modulating interventions are applied repeatedly or over extended periods, although direct functional or injury-related implications cannot be inferred from histomorphological data alone. However, it remains unclear whether repeated administration of a TRPA1 antagonist affects skeletal muscle or muscle spindle morphology under healthy, resting conditions.

Therefore, this exploratory study aimed to determine whether repeated systemic exposure to the TRPA1 antagonist HC-030031 is associated with detectable histomorphological alterations in skeletal muscle fibers and muscle spindles in healthy young rats. We hypothesized that repeated HC-030031 administration would not be associated with detectable changes in muscle mass or morphology of extrafusal and intrafusal fibers under resting conditions. To test this hypothesis, we performed quantitative histological and morphometric analyses of the tibialis anterior muscle after four weeks of repeated administration of a TRPA1 antagonist to young adult rats.

## 2. Results

### 2.1. Body and Muscle Mass

All variables analyzed using the unpaired Student’s *t*-test satisfied the normality assumption based on the Shapiro–Wilk test. The age at sampling did not differ significantly between the control (Con) and HC-030031-treated (HC) groups (Table 1). No significant differences were observed in body weight between the Con and HC groups. Similarly, the tibialis anterior (TA) muscle mass and TA muscle mass relative to body mass did not differ significantly between groups (Table 1).

### 2.2. Morphological Characteristics of Extrafusal Muscle Fibers

**Figure 1:**
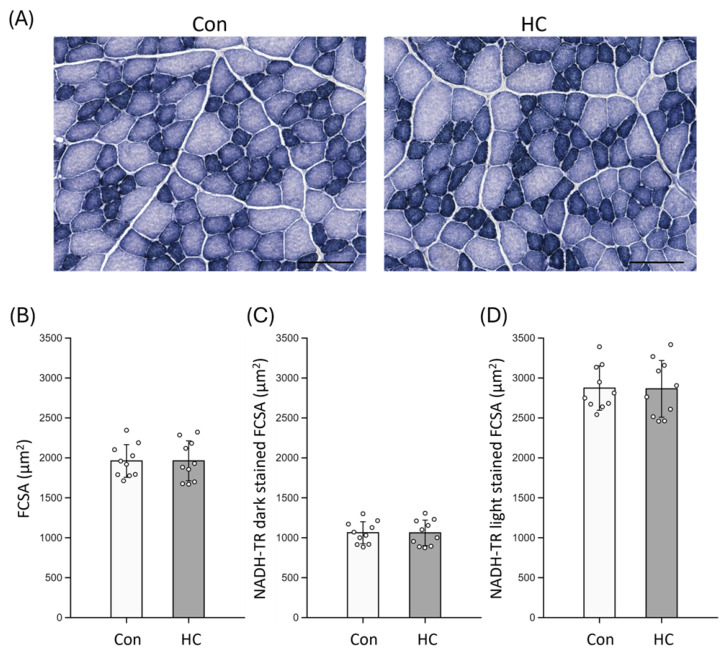
Representative NADH-TR-stained cross-sectional images of the tibialis anterior muscle in the control (Con) and HC-030031-treated (HC) rats, showing dark- and light-stained fibers reflecting differences in oxidative enzyme activity (**A**), FCSA of all extrafusal muscle fibers (**B**), FCSA of NADH-TR dark-stained fibers (**C**), and FCSA of NADH-TR light-stained fibers (**D**). The scale bar represents 100 μm. Values are expressed as mean ± SD with individual data points overlaid, and no significant differences were observed between groups. Each data point represents one animal; *n* = 10 rats per group.

NADH-TR staining was used as a histochemical indicator of oxidative enzyme activity, allowing the visualization of differences in oxidative staining intensity among muscle fibers. Representative NADH-TR-stained cross-sections of the TA muscle are shown in Figure 1A and exhibit a typical mosaic pattern of NADH-TR dark- and light-stained fibers. Quantitative analysis revealed no significant differences between the Con and HC groups in the mean FCSA of all extrafusal muscle fibers (Figure 1B) or the myofiber roundness (Table 1). To further examine fiber morphology according to the oxidative staining intensity, dark- and light-stained fibers were analyzed separately based on grayscale intensity values. This analysis was not intended to define the conventional myosin heavy chain-based fiber types. No significant differences were observed in the FCSA of NADH-TR dark-stained (Figure 1C) or light-stained fibers (Figure 1D) between the Con and HC groups.

ANCOVA revealed a significant effect of age on FCSA (F (1, 17) = 29.52, *p* = 4.245 × 10^−5^), reflecting the natural muscle growth. However, after adjusting for age, there was no significant difference in FCSA between the Con and HC groups (F (1, 17) = 0.1612, *p* = 0.693), suggesting that the 4-week administration of HC-030031 did not affect the muscle fiber size.

### 2.3. Morphological Characteristics of Muscle Spindles and Intrafusal Fibers

**Figure 2:**
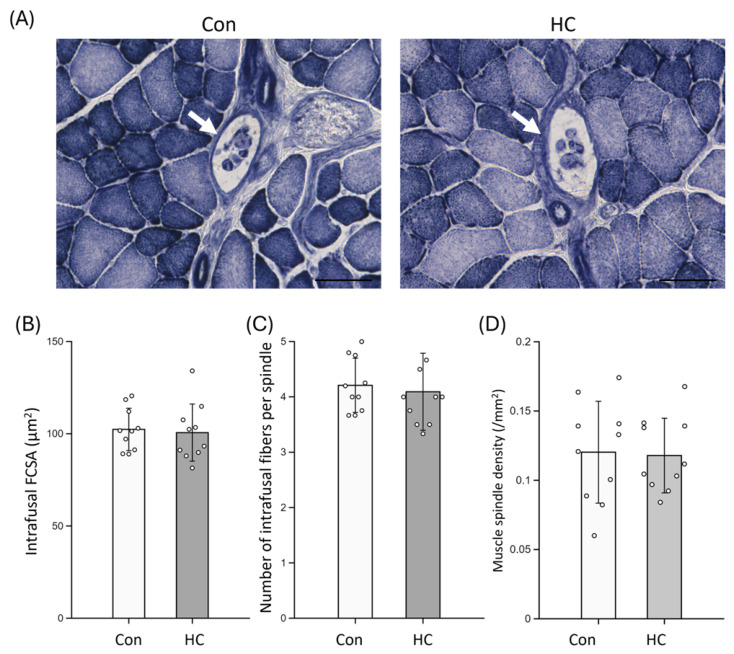
Representative NADH-TR-stained cross-sectional images of muscle spindles (arrows) in the tibialis anterior muscle from the control (Con) and HC-030031-treated (HC) rats, in which intrafusal fibers enclosed within a connective tissue capsule are distinguishable from surrounding extrafusal fibers (**A**), FCSA of intrafusal muscle fibers (**B**), number of intrafusal fibers per muscle spindle (**C**), and muscle spindle density (**D**). The scale bar represents 50 μm. Values are expressed as mean ± SD with individual data points overlaid, and no significant differences were observed between groups. Each data point represents one animal; *n* = 10 rats per group.

Representative images of the muscle spindles are shown in Figure 2A. Muscle spindles were identifiable in both groups, with intrafusal fibers enclosed within a connective tissue capsule and distinguishable from the surrounding extrafusal fibers. Quantitative analysis revealed no significant differences in the FCSA of intrafusal muscle fibers (Figure 2B) or intrafusal fiber roundness (Table 1) between the Con and HC groups. In addition, the number of intrafusal fibers per muscle spindle (Figure 2C) and muscle spindle density (Figure 2D) were not significantly different between the groups.

Overall, the 95% confidence intervals for the measured morphometric outcomes generally included zero, supporting the absence of detectable group differences under these experimental conditions.

## 3. Discussion

This study examined whether repeated HC-030031 administration affected the histomorphology of skeletal muscle fibers and muscle spindles in male rats. The principal finding was that four weeks of repeated HC-030031 administration was not associated with detectable changes in muscle wet weight, extrafusal FCSA, or intrafusal muscle fiber morphology in the tibialis anterior muscle. These results indicate that repeated HC-030031 administration did not induce detectable structural alterations in the skeletal muscle or muscle spindle architecture under the experimental conditions examined.

NADH-TR staining was performed to examine whether repeated HC-030031 administration was associated with gross alterations in the oxidative staining patterns of skeletal muscle fibers. Because sensory signaling, inflammation, and muscle activity can influence the skeletal muscle metabolic phenotype, the assessment of oxidative enzyme staining provides useful complementary information to morphometric analysis. In the present study, the FCSA of dark- and light-stained fibers did not differ between the Con and HC groups, suggesting that repeated HC-030031 administration was not associated with detectable changes in fiber size within either staining intensity-based subset. However, this analysis was not designed to provide a comprehensive fiber-type classification or a quantitative assessment of oxidative metabolism. Therefore, NADH-TR findings should be interpreted as staining intensity-based morphometric observations rather than evidence of preserved mitochondrial function or metabolic capacity. Future studies using quantitative analyses of oxidative enzyme activity, mitochondrial markers, and fiber composition are necessary to determine whether repeated HC-030031 administration affects the skeletal muscle’s metabolic phenotype.

An important consideration when interpreting the present findings is that the pharmacological efficacy of HC-030031 was not directly verified in experimental animals. Although the dosing regimen was selected based on previous studies demonstrating effective suppression of TRPA1-mediated nociceptive responses in rodents, direct confirmation of TRPA1 inhibition was not performed in this study. Therefore, the present results should be interpreted as evidence that repeated HC-030031 administration did not induce detectable histomorphological alterations under the experimental conditions used.

Although no significant morphological differences were detected, these findings may provide useful information regarding tissue integrity during prolonged exposure to TRPA1 antagonists. TRPA1 channels are widely expressed in nociceptive sensory neurons and are known to contribute to mechanical hyperalgesia and inflammatory pain signaling [12,13]. Because TRPA1 antagonists have been investigated as potential therapeutic agents for pain modulation, evaluating their potential effects on peripheral musculoskeletal tissues is an important consideration. In this regard, the absence of detectable alterations in extrafusal and intrafusal muscle morphology suggests that repeated HC-030031 administration was not associated with overt structural changes in healthy skeletal muscle of male rats under the conditions examined.

The structural maintenance of intrafusal muscle fibers is believed to depend on complex trophic interactions between muscle spindles and their associated neural inputs. Intrafusal fibers are innervated by γ-motor neurons and receive sensory input from group Ia and II afferents, forming an integrated proprioceptive system that regulates muscle length and stretch sensitivity [11,14]. Previous studies have shown that peripheral nerve injury or denervation can induce structural changes in muscle spindles, including reductions in intrafusal fiber size and alterations in spindle morphology [15,16,17]. These findings suggest that neural activity and trophic signaling are important determinants of spindle structural maintenance. Neurotrophic factors, such as neurotrophin-3, also contribute to the development and maintenance of muscle spindle structure through interactions with proprioceptive afferents, although these mechanisms are primarily associated with proprioceptive rather than nociceptive pathways [18,19,20].

In contrast to these proprioceptive pathways, TRPA1 is predominantly expressed in nociceptive sensory neurons, particularly in small-diameter Aδ and C fibers involved in pain and inflammatory signaling [12,13]. Because TRPA1 expression is largely associated with nociceptive afferents rather than proprioceptive or fusimotor neurons, TRPA1-associated nociceptive pathways are functionally distinct from the neural pathways involved in muscle spindle structural maintenance. The absence of morphological changes in intrafusal fibers observed in the present study is consistent with the functional organization of peripheral sensory systems. It should also be noted that the present study did not distinguish nuclear bag and nuclear chain fibers because the primary objective was to evaluate overall muscle spindle morphology rather than individual intrafusal fiber subtypes. Future studies employing subtype-specific analyses may provide additional insight into whether repeated HC-030031 administration differentially affects individual intrafusal fiber populations.

An additional consideration is that the present study did not directly evaluate behavioral or sensory responses to confirm functional inhibition of TRPA1 signaling in vivo. Consequently, pharmacological efficacy was inferred from the dosing regimen rather than directly verified through nociceptive assays. The dose of HC-030031 used in this study was selected with reference to a previous rodent study reporting that systemic HC-030031 administration attenuated TRPA1-related nociceptive responses at doses within a similar range [21]. However, differences in the administration route, dosing frequency, and experimental endpoints should be considered when extrapolating from these previous studies to the present experimental design. Therefore, future studies incorporating TRPA1-dependent behavioral assays or electrophysiological measurements are necessary to confirm the pharmacological efficacy under the present dosing conditions and to further clarify the relationship between repeated HC-030031 administration and the sensory physiology of muscle.

Another important limitation of the present study is the administration schedule. The present protocol should be interpreted as an intermittent repeated exposure protocol rather than a model of continuous pharmacological TRPA1 blockade. HC-030031 was administered once weekly for four weeks to examine whether four intermittent systemic exposures to the antagonist were associated with detectable histomorphological alterations in skeletal muscle fibers or muscle spindles under healthy, resting conditions. The 4-week period was selected as an exploratory time frame because skeletal muscle morphological changes, including alterations in muscle mass, myofiber size, and subcellular architecture, can be detected within several weeks in rodent models of altered neuromuscular activity or disuse [22,23]. However, the duration of pharmacological action after each injection was not determined, and the dosing interval and treatment duration were not specifically optimized to determine the time course of HC-030031-related effects on skeletal muscle or muscle spindle morphology. The inclusion of a positive control condition would have further strengthened the interpretation of the present findings. Such a control would have been helpful for verifying pharmacological efficacy through a TRPA1-dependent sensory or behavioral response or for confirming the sensitivity of the histological and morphometric procedures using a condition known to induce skeletal muscle or muscle spindle remodeling. Therefore, the present findings should be interpreted within the limits of this experimental design, and future studies incorporating appropriate positive control conditions are warranted.

The present study had several limitations. First, the analyses focused on the histomorphological characteristics of muscle fibers and did not directly evaluate the functional properties of muscle spindles, such as afferent discharge and spindle sensitivity. Although structural alterations are often associated with functional changes, preserved morphology does not necessarily exclude subtle physiological modifications in proprioceptive signaling [11]. Second, the age of the rats at sampling ranged from 10 to 17 weeks, covering the transition from late adolescence to early adulthood. As muscle fiber size can change during this developmental period, we performed an analysis of covariance (ANCOVA) to account for the potential influence of age. Although age was significantly associated with fiber cross-sectional area (FCSA), no significant group differences were detected after adjusting for age (F (1, 17) = 0.1612, *p* = 0.693). These results suggest that the absence of a detectable effect of HC-030031 administration on extrafusal muscle fiber size was not due to the age range of the animals. Third, the present experiments were performed using healthy rats under physiological conditions. Furthermore, this study evaluated only male rats, and potential sex-specific differences in TRPA1 signaling and muscle morphology remain to be explored in future investigations. As TRPA1 plays a prominent role in inflammatory and nociceptive signaling, its inhibition may produce different outcomes in pathological states involving muscle injury or inflammation. Further studies incorporating disease models or different administration periods may be beneficial to fully clarify the long-term systemic effects of TRPA1 modulation in various physiological contexts.

Despite these limitations, the present study provides histological evidence that, under the present intermittent repeated-administration protocol, HC-030031 was not associated with detectable histomorphological alterations in the skeletal muscle fibers or muscle spindles in healthy young male rats. These findings contribute to the understanding of repeated TRPA1 antagonist exposure in skeletal muscles; however, further functional and pathological studies are needed before broader physiological or applied implications can be inferred.

## 4. Materials and Methods

### 4.1. Animals and Ethical Approval

Twenty male Fischer 344 rats (CLEA, Tokyo, Japan) were used in this study. Rats (*n* = 20, mean age at the start of the experiment, 8.9 ± 3.4 weeks) were housed under controlled environmental conditions (23 ± 2 °C, 55 ± 10% humidity, and a 12-h light/dark cycle) with free access to CE-2 rodent chow and water. Young rats spanning late adolescence to early adulthood were included because the present study aimed to examine the effects of repeated HC-030031 administration on skeletal muscle and muscle spindle morphology under healthy, resting conditions. As skeletal muscle maturation has been reported to continue across this age range in rats [24], the age at sampling was considered in the statistical analysis of the extrafusal fiber cross-sectional area. The sample size was determined a priori using G*Power software (version 3.1.9.7) for an unpaired *t*-test. Based on myofiber cross-sectional area (FCSA) data obtained in previous studies from our laboratory [25], a minimum sample size of 10 rats per group was estimated to detect a significant between-group difference with an α level of 0.05 and a statistical power of 0.80. All procedures were approved by the Institutional Animal Care and Use Committee of the National Institute of Fitness and Sports in Kanoya (R5-03) and conducted in accordance with the Guiding Principles for the Care and Use of Animals in the Field of Physiological Sciences published by the Physiological Society of Japan available online: https://www.physiology.jp/ctrl-seiri/wp-content/uploads/2015/07/animal-guideline-20150302.pdf; accessed on 17 June 2026).

### 4.2. Experimental Design and TRPA1 Antagonist Administration

Rats were randomly assigned to two groups: a control group receiving the vehicle (Con, *n* = 10) and an HC-030031-treated group (HC, *n* = 10). HC-030031 (Cosmo Bio Co., Tokyo, Japan), a TRPA1 antagonist, was administered to the HC group. HC-030031 was dissolved in dimethyl sulfoxide (DMSO; FUJIFILM Wako Pure Chemical Corp., Osaka, Japan) before use. The HC group received subcutaneous injections of HC-030031 once a week for four consecutive weeks. The initial dose was 50 mg/kg body weight, followed by 100 mg/kg in the subsequent injections. This dosing range was selected with reference to a previous rodent study reporting that systemic HC-030031 administration attenuated TRPA1-related nociceptive response at doses within a similar range [21]. The control group received an equivalent volume of vehicle (DMSO) according to the same schedule as the treatment groups. All injections were performed at approximately the same time of the day to minimize potential circadian effects.

### 4.3. Muscle Sampling and Tissue Preparation

After the 4-week intervention, the rats were anesthetized by isoflurane inhalation (2.0–2.5%), and the left TA muscle was excised. After removal of excess connective tissue, the wet muscle weight was measured. The central one-third portion of the muscle belly was trimmed and mounted on a piece of cork with an optimal cutting temperature (OCT) compound. Samples were frozen in isopentane cooled in liquid nitrogen for histological analyses and stored at −80 °C until further processing. Serial transverse cryosections (10 µm thickness) were prepared from the frozen tissue blocks using a cryostat (CM3050S, Leica, Germany) maintained at −20 °C for subsequent histochemical analyses. All muscles were embedded with the longitudinal axis oriented perpendicular to the cutting plane, and transverse cryosections were obtained from the mid-belly region. Representative sections were inspected to confirm the absence of systematic elongation of extrafusal or intrafusal fibers indicative of oblique sectioning.

### 4.4. Histochemical Analyses

Nicotinamide adenine dinucleotide-tetrazolium reductase (NADH-TR) staining was performed on transverse cryosections to evaluate oxidative enzyme activity and muscle fiber oxidative capacity. Briefly, 10-µm-thick cryosections were air-dried at room temperature and rinsed in 0.05 M Tris-HCl buffer (pH 7.4). Sections were then incubated at 37 °C for 30 min in a reaction solution consisting of 0.05 M Tris-HCl buffer (pH 7.4) supplemented with nitro blue tetrazolium (NBT, 1.2 mM, Kanto Chemical Co., Inc., Tokyo, Japan) and β-nicotinamide adenine dinucleotide (β-NADH, 1.1 mM, Sigma-Aldrich, Tokyo, Japan) to visualize oxidative activity, according to standard histochemical procedures [26,27]. After incubation, sections were rinsed in distilled water and differentiated by sequential immersion in graded acetone solutions (30, 60, 90, 60, and 30% *v*/*v*) to remove nonspecific background and excess lipids. Sections were finally washed in distilled water and coverslipped using an aqueous mounting medium.

### 4.5. Morphometric Analysis

Digital images were acquired at 200-fold magnification using a light/fluorescence microscope (BX60; Olympus, Tokyo, Japan) equipped with a charge-coupled device (CCD) camera (DP73; Olympus) for subsequent morphometric analysis [25,28]. Morphometric analyses were performed using Image-Pro Premier software version 10.0 (Media Cybernetics, Rockville, MD, USA). The FCSA and roundness of the extrafusal muscle fibers were quantified based on NADH-TR-stained sections. For each muscle, the FCSAs of at least 100 muscle fibers were measured to obtain representative values [29]. Transverse sections derived from the central portion of the muscle belly were analyzed. Within each section, five predefined regions (central, superficial, deep, anterior, and posterior areas) were selected to obtain representative morphometric data.

All muscle fibers within the selected regions were traced and analyzed without pre-selection. Grayscale intensity values ranging from 0 to 255 were obtained for each fiber from digitized NADH-TR-stained images using Image-Pro software. For the purpose of examining whether HC-030031 administration affected fiber size according to NADH-TR staining intensity, dark- and light-stained fibers were analyzed separately. The FCSA of each staining-based fiber subset was compared between the Con and HC groups. In the analyzed images, dark-stained fibers exhibited lower grayscale values (49 ± 5; range, 41–58), whereas light-stained fibers exhibited higher grayscale values (120 ± 6; range, 105–130). This separation was based on NADH-TR staining intensity and was not intended to represent conventional myosin heavy chain-based fiber type classification.

Muscle spindles were identified based on established morphological criteria, including a fusiform connective tissue capsule surrounding a group of small intrafusal muscle fibers that were clearly distinguishable from adjacent extrafusal fibers [30,31]. The FCSA and roundness of the intrafusal and extrafusal muscle fibers were quantified. Roundness was analyzed as an index of fiber shape and was calculated as (perimeter^2^)/(4π × FCSA), where perimeter represents the fiber perimeter and FCSA represents the myofiber cross-sectional area. Muscle spindle density was calculated as the number of muscle spindles divided by the whole muscle cross-sectional area (mm^2^).

### 4.6. Statistical Analysis

No animals or data points were excluded, and all collected data were included in the final analysis. All data are presented as the mean ± standard deviation (SD). The results are also presented as mean differences with 95% confidence intervals (CI). The normality of the data distribution was assessed using the Shapiro–Wilk test. All variables satisfied the normality assumption; therefore, comparisons between the two groups were performed using an unpaired Student’s *t*-test, using the SPSS Statistical Package Version 25.0 (IBM, Chicago, IL, USA). To assess the magnitude of the differences between the two groups, Cohen’s *d* was calculated as the effect size for unpaired *t*-tests. To compare the mean values of the FCSA between the Con and HC groups while accounting for the potential influence of the age at sampling, an analysis of covariance (ANCOVA) was performed using a multiple linear regression model in GraphPad Prism (version 9.0; GraphPad Software, San Diego, CA, USA). In this model, FCSA was defined as the dependent variable, group (Con vs. HC) as the independent variable, and age as the covariate. The assumption of parallelism was verified by confirming that the interaction between group and age was not significant, indicating that the relationship between age and FCSA was consistent across both groups. The primary outcome measure was the FCSA of extrafusal muscle fibers. A *p*-value of less than 0.05 was considered statistically significant.

## 5. Conclusions

In conclusion, under the present intermittent repeated-administration protocol, HC-030031 was not associated with detectable histomorphological alterations in extrafusal muscle fibers or intrafusal muscle spindle components in the tibialis anterior muscle of healthy young male rats. Future studies incorporating TRPA1-dependent functional assays, proprioceptive assessments, and evaluations under conditions of exercise, muscle injury, or pathological sensory alterations are necessary to further elucidate the physiological and applied implications of TRPA1-targeted interventions.

## Figures and Tables

**Table 1:** Age and morphometric characteristics of extrafusal and intrafusal muscle fibers and muscle spindle profiles in the tibialis anterior (TA) muscle. Values are presented as mean ± SD. Mean differences with 95% confidence intervals (CI) and Cohen’s *d* are presented. Statistical analysis was performed using an unpaired *t*-test. Con, control (*n* = 10); HC, HC-030031-treated (*n* = 10). CSA, whole muscle cross-sectional area. FCSA, myofiber cross-sectional area.

	Con (*n* = 10)	HC (*n* = 10)	Mean Difference (95% CI)	*p*-Value	Cohen’s *d*
Age (weeks)	12.6 ± 3.3	13.2 ± 3.7	0.51 [−2.79, 3.81]	0.749	0.145
TA muscle mass (mg)	454 ± 99	425 ± 80	−28.70 [−113.31, 55.91]	0.485	0.319
TA muscle mass/body mass (mg/g)	1.86 ± 0.25	1.78 ± 0.10	−0.08 [−0.26, 0.11]	0.374	0.414
TA CSA (mm^2^)	35.5 ± 7.6	32.4 ± 3.8	3.03 [−8.69, 2.63]	0.188	0.504
Extrafusal muscle fibers					
Extrafusal FCSA (μm^2^)	1962 ± 205	1963 ± 251	0.85 [−214.18, 215.88]	0.993	0.004
Extrafusal myofiber roundness	1.21 ± 0.01	1.22 ± 0.01	0.01 [−0.01, 0.01]	0.483	0.336
NADH-TR dark FCSA (μm^2^)	1063 ± 139	1061 ± 159	−1.34 [−141.96, 139.28]	0.984	0.009
NADH-TR light FCSA (μm^2^)	2872 ± 276	2865 ± 354	−6.93 [−305.25, 291.39]	0.962	0.022
Intrafusal muscle fibers					
Intrafusal FCSA (μm^2^)	102 ± 11	100 ± 16	−1.77 [−14.61, 11.06]	0.775	0.130
Intrafusal fiber roundness	1.13 ± 0.03	1.16 ± 0.05	0.03 [−0.01, 0.07]	0.071	0.878
Muscle spindle profiles					
Number of intrafusal fibers per muscle spindle	4.2 ± 0.5	4.0 ± 0.8	−0.12 [−0.68, 0.45]	0.671	0.193
Muscle spindle density (/mm^2^)	0.12 ± 0.04	0.12 ± 0.03	−0.00 [−0.03, 0.03]	0.867	0.076

## Data Availability

The original contributions presented in this study are included in the article. Further inquiries can be directed to the corresponding author.
